# Contribution of the C-terminal tri-lysine regions of human immunodeficiency virus type 1 integrase for efficient reverse transcription and viral DNA nuclear import

**DOI:** 10.1186/1742-4690-2-62

**Published:** 2005-10-18

**Authors:** Zhujun Ao, Keith R Fowke, Éric A Cohen, Xiaojian Yao

**Affiliations:** 1 Laboratory of Molecular Human Retrovirology, Faculty of Medicine, University of Manitoba, Winnipeg, Manitoba R3E 0W3, Canada; 2 Department of Medical Microbiology, Faculty of Medicine, University of Manitoba, Winnipeg, Manitoba R3E 0W3, Canada; 3 Laboratory of Human Retrovirology, Institut de Recherches Cliniques de Montréal, Département de microbiologie et immunologie, Faculté de Médecine, Université de Montréal, Montréal, Quebec H2W 1R7, Canada

## Abstract

**Background:**

In addition to mediating the integration process, HIV-1 integrase (IN) has also been implicated in different steps during viral life cycle including reverse transcription and viral DNA nuclear import. Although the karyophilic property of HIV-1 IN has been well demonstrated using a variety of experimental approaches, the definition of domain(s) and/or motif(s) within the protein that mediate viral DNA nuclear import and its mechanism are still disputed and controversial. In this study, we performed mutagenic analyses to investigate the contribution of different regions in the C-terminal domain of HIV-1 IN to protein nuclear localization as well as their effects on virus infection.

**Results:**

Our analysis showed that replacing lysine residues in two highly conserved tri-lysine regions, which are located within previously described Region C (^235^WKGPAKLLWKGEGAVV) and sequence Q (^211^KELQKQITK) in the C-terminal domain of HIV-1 IN, impaired protein nuclear accumulation, while mutations for RK_263,4 _had no significant effect. Analysis of their effects on viral infection in a VSV-G pseudotyped RT/IN trans-complemented HIV-1 single cycle replication system revealed that all three C-terminal mutant viruses (KK215,9AA, KK240,4AE and RK263,4AA) exhibited more severe defect of induction of β-Gal positive cells and luciferase activity than an IN class 1 mutant D64E in HeLa-CD4-CCR5-β-Gal cells, and in dividing as well as non-dividing C8166 T cells, suggesting that some viral defects are occurring prior to viral integration. Furthermore, by analyzing viral DNA synthesis and the nucleus-associated viral DNA level, the results clearly showed that, although all three C-terminal mutants inhibited viral reverse transcription to different extents, the KK240,4AE mutant exhibited most profound effect on this step, whereas KK215,9AA significantly impaired viral DNA nuclear import. In addition, our analysis could not detect viral DNA integration in each C-terminal mutant infection, even though they displayed various low levels of nucleus-associated viral DNA, suggesting that these C-terminal mutants also impaired viral DNA integration ability.

**Conclusion:**

All of these results indicate that, in addition to being involved in HIV-1 reverse transcription and integration, the C-terminal tri-lysine regions of IN also contribute to efficient viral DNA nuclear import during the early stage of HIV-1 replication.

## Background

The integrase (IN) of human immunodeficiency virus type 1 (HIV-1) is encoded by the *pol *gene and catalyzes integration of viral cDNA into host chromosome, an essential step in HIV-1 replication. In addition to mediating the integration process, HIV-1 IN also participates in different steps during viral life cycle, including reverse transcription and viral DNA nuclear import [1-6]. During early phase of the HIV-1 replication cycle, after virus entry into target cells, another *pol *gene product, reverse transcriptase (RT), copies viral genomic RNA into double-stranded cDNA which exists within a nucleoprotein preintegration complex (PIC). The PIC also contains viral proteins including RT, IN, nucleocapsid (NC, p9), Vpr and matrix (MA, p17) and this large nucleoprotein complex is capable of actively translocating into the cell nucleus, including that of non-dividing cells (reviewed in reference [7]). This feature is particularly important for the establishment of HIV-1 replication and pathogenesis in exposed hosts, since the infection of postmitotic cells including tissue macrophages, mucosal dendritic cells as well as non-dividing T cells may be essential not only for viral transmission and dissemination, but also for the establishment of persistent viral reservoirs.

HIV-1 IN is composed of three functional domains, an N-terminal domain, a central catalytic core domain and a C-terminal domain, all of which are required for a complete integration reaction. The N-terminal domain harbors an HHCC-type zinc binding domain and is implicated in the multimerization of the protein and contributes to the specific recognition of DNA ends [8-10]. The core domain of IN contains the highly conserved DDE motif which is important for catalytic activity of the protein [11,12]. The C-terminal domain was shown to possess nonspecific DNA binding properties [13,14]. Some mutations within this region cause a drastic loss of virus infectivity without affecting the enzymatic activity of IN *in vitro *[2,13-16]. There are three conserved sequences in the C-terminus of IN that are essential for HIV-1 replication. Regions C (^235^WKGPAKLLWKGEGAVV) and N (^259^VVPRRKAK) are conserved in all known retroviruses and the ^211^KELQKQITK motif falls within the so-called glutamine-rich based region (sequence Q) of lentiviruses [17]. Alteration of each of the three sequences such as Q214L/Q216L, K215A/K219A, W235E, K236A/K240A, K244A/E246A, RRE263-5AAH resulted in loss of viral replication [15-18]. However, the mechanism(s) underlying the loss of viral infectivity remains controversial.

A number of studies have demonstrated the karyophilic properties of IN implicating that this protein may play an important role for PIC nuclear import [3,19-23]. However, the definition of nuclear localization signals (NLSs) in IN as well as their contribution to HIV-1 PIC nuclear import still remain to be determined. Previous report has suggested an atypical bipartite NLS (^186^KRK and ^211^KELQKQITK) by showing that IN mutants K186Q and Q214/216L in these regions lost the protein nuclear localization and their inability to bind to karyopherin α *in vitro *[3]. However, in attempt to analyze the effect of these mutants during HIV-1 replication, other studies did not reveal the importance of these IN mutants (K186Q and Q214/216L) for viral nuclear import; rather they appear to be required for reverse transcription, integration or undefined post-nuclear entry steps [16,18,23]. Also, another IN amino acid sequence IIGQVRDQAEHLK (aa161–173), was initially identified as an atypical NLS, which is required for viral DNA nuclear import [19]. However, reassessments of this putative NLS function failed to confirm this conclusion [24,25]. Some reports have also acknowledged that IN localization could result from passive diffusion of the protein and its DNA binding property [26,27], but DNA binding alone does not fully explain a rapid, ATP- and temperature-dependent nuclear import of IN [20]. It has recently been reported that the nuclear translocation of HIV-1 IN can be attributed to its interaction with a cellular component, human lens epithelium-derived growth factor/transcription coactivator p75 (LEDGF/p75) and LEDGF/p75 was also shown to be a component of HIV PIC [28,29]. However, whether this IN/LEDGF/p75 interaction plays an important role for HIV-1 nuclear import still remains to be elucidated, since HIV-1 infection and replication in LEDGF/p75-deficient cells was equivalent to that in control cells, regardless whether cells were dividing or growth arrested [29]. Thus, even though extensive studies have been dedicated in this specific research field, the contribution of HIV-1 IN to viral PIC nuclear import remains to be defined.

In this study, we have performed substitution mutational analysis to investigate the contribution of different C-terminal regions of IN to protein nuclear localization and their effects on HIV-1 replication. Our results showed that mutations of lysine residues in two tri-lysine regions, which are located within previously described Region C and sequence Q [17] in the C-terminal domain of HIV-1 IN, impaired protein nuclear localization, while mutations of arginines at amino acid position of 263 and 264 in the distal part of the C-terminal domain of IN had no significant effect. Moreover, we assessed the effect of these IN mutants during HIV-1 single cycle infection mediated by VSV-G pseudotyped RT/IN trans-complemented viruses. Results showed that, while all three C-terminal mutant viruses differentially affected HIV-1 reverse transcription, the KK240,4AE mutant exhibited most profound inhibition on this step, whereas KK215,9AA significantly impaired viral DNA nuclear import.

## Results

### The C-terminal domain of HIV-1 integrase (IN) is required for the nuclear localization of IN-YFP fusion protein

**Figure 1:**
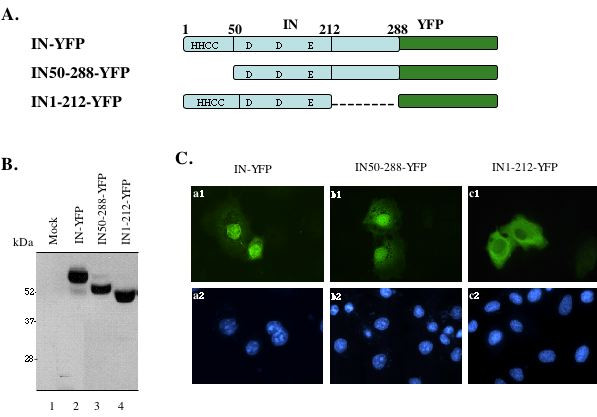
**Subcellular localization of the wild-type and truncated HIV integrase fused with YFP**. A) Schematic structure of HIV-1 integrase-YFP fusion proteins. Full-length (1–288aa) HIV-1 integrase, the N-terminus-truncated mutant (51–228aa) or the C-terminus-truncated mutant (1–212aa) was fused in frame at the N-terminus of YFP protein. The cDNA encoding for each IN-YFP fusion protein was inserted in a SVCMV expression plasmid. B) Expression of different IN-YFP fusion proteins in 293T cells. 293T cells were transfected with each IN-YFP expressor and at 48 hours of transfection, cells were lysed, immunoprecipitated with anti-HIV serum and resolved by electrophoresis through a 12.5% SDS-PAGE followed by Western blot with rabbit anti-GFP antibody. The molecular weight markers are indicated at the left side of the gel. C) Intracellular localization of different IN-YFP fusion proteins. HeLa cells were transfected with each HIV-1 IN-YFP fusion protein expressor and at 48 hours of transfection, cells were fixed and subjected to indirect immunofluorescence using rabbit anti-GFP and then incubated with FITC-conjugated anti-rabbit antibodies. The localization of each fusion protein was viewed by Fluorescence microscopy with a 50× oil immersion objective. Upper panel is fluorescence images and bottom panel is DAPI nucleus staining.

**Figure 2:**
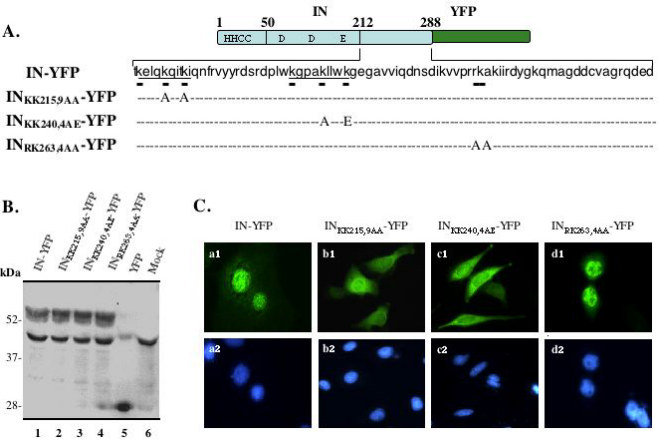
**Effect of different IN C-terminal substitution mutants on IN-YFP intracellular localization**. A) Diagram of HIV-1 IN domain structure and introduced mutations at the C-terminal domain of the protein. The position of lysines in two tri-lysine regions and introduced mutations are shown at the bottom of sequence. B) The expression of the wild-type and mutant IN-YFP fusion proteins were detected in transfected 293T cells by using immunoprecipitation with anti-HIV serum and Western blot with rabbit anti-GFP antibody, as described in figure 1. The molecular weight markers are indicated at the left side of the gel. C) Intracellular localization of different HIV-1 IN mutant-YFP fusion proteins in HeLa cells were analyzed by fluorescence microscopy with a 50× oil immersion objective. The nucleus of HeLa cells was simultaneously visualized by DAPI staining (lower panel).

In this study, we first investigated the intracellular localization of HIV-1 IN and delineated the region(s) of IN contributing to its karyophilic property. A HIV-1 IN-YFP fusion protein expressor (CMV-IN-YFP) was generated by fusing a full-length HIV-1 IN cDNA (amplified from HIV-1 HxBru molecular clone [30]) to the 5' end of YFP cDNA in a CMV-IN-YFP expressor, as described in Materials and Methods. Transfection of CMV-IN-YFP expressor in 293T cells resulted in the expression of a 57 kDa IN-YFP fusion protein (Fig. 1B, lane 2; Fig. 2B, lane 1), whereas expression of YFP alone resulted in a 27 kDa protein (Fig. 2B, lane 5). Given that HeLa cells have well-defined morphology and are suitable for observation of intracellular protein distribution, we tested the intracellular localization of YFP and IN-YFP by transfecting CMV-IN-YFP or CMV-YFP expressor in HeLa cells. After 48 hours of transfection, cells were fixed and subjected to indirect immunofluorescence assay using primary rabbit anti-GFP antibody followed by secondary FITC-conjugated anti-rabbit antibodies. Results showed that, in contrast to a diffused intracellular localization pattern of YFP (data not shown), the IN-YFP fusion protein was predominantly localized in the nucleus (Fig 1C, a1), confirming the karyophilic feature of HIV-1 IN.

To delineate the karyophilic determinant in HIV-1 IN, two truncated IN-YFP expressors CMV-IN_50–288_-YFP and CMV-IN_1–212_-YFP were generated. In CMV-IN_50–288_-YFP, the N-terminal HH-CC domain of IN (aa 1–49) was deleted and in CMV-IN_1–212_-YFP, the C-terminal domain (aa 213–288) was removed (Fig. 1A). Transfection of each truncated IN-YFP fusion protein expressor in 293T cells resulted in the expression of IN_50–288_-YFP and IN_1–212_-YFP at approximately 52 kDa and 48 kDa molecular mass respectively (Fig. 1B, lanes 3 and 4). We next investigated the intracellular localization of truncated IN-YFP fusion proteins in HeLa cells by using indirect immunofluorescence assay, as described above. Results showed that the IN_50–288_-YFP was predominantly localized in the nucleus with a similar pattern as the wild-type IN-YFP fusion protein (Fig. 1C, compare b1 to a1). However, IN_1–212_-YFP fusion protein was excluded from the nucleus, with an accumulation of the mutant protein in the cytoplasm (Fig 1C, c1). These results were also further confirmed by using rabbit anti-IN antibody immunofluorescence assay (data not shown). Taken together, our data show that the C-terminal domain of HIV-1 IN is required for its nuclear accumulation.

### Two tri-lysine regions in the C-terminal domain of IN are involved in the protein nuclear localization

The C-terminal domain of HIV-1 IN contains several regions that are highly conserved in different HIV-1 strains, including Q, C and N regions [17]. Interestingly, in regions Q and C, sequences of ^211^KELQKQITK and ^236^KGPAKLLWK possess high similarity in terms of numbers and position of lysine residues and therefore, we term them proximal tri-lysine region and distal tri-lysine region, respectively (Fig. 2A). All of these lysine residues are highly conserved in most HIV-1 strains [31]. To test whether these basic lysine residues could constitute for a possible nuclear localization signal for IN nuclear localization, we specifically introduced substitution mutations for two lysines in each tri-lysine region and generated IN_KK215,9AA_-YFP and IN_KK240,4AE_-YFP expressors (Fig. 2A). In the conserved N region, there is a stretch of four basic residues among five amino acids (aa) ^262^RRKAK. To characterize whether this basic aa region may contributes to IN nuclear localization, we replaced an arginine and a lysine at positions of 263 and 264 by alanines in this region and generated a mutant (IN_RK263,4AA_-YFP). The protein expression of different IN-YFP mutants in 293T cells showed that, like the wild type IN-YFP, each IN-YFP mutant fusion protein was detected at similar molecular mass (57 kDa) in SDS-PAGE (Fig 2B, lanes 1 to 4), while YFP alone was detected at position of 27 kDa (lane 5). Then, the intracellular localization of each IN mutant was investigated in HeLa cells by using similar methods, as described above. Results showed that, while the wild type IN-YFP and IN_RK263,4AA_-YFP still predominantly localized to the nucleus (Fig. 2C, a1 and d1), both IN_KK215,9AA_-YFP and IN_KK240,4AE_-YFP fusion proteins were shown to distribute throughout the cytoplasm and nucleus, but with much less intensity in the nucleus (Fig 2C, a1 and b1). These data suggest that these lysine residues in each tri-lysine regions are required for efficient HIV-1 IN nuclear localization.

### Production of VSV-G pseudotyped HIV-1 IN mutant viruses and their effects on HIV-1 infection

**Figure 3:**
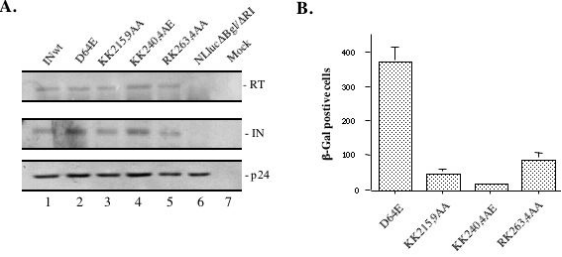
**Production of different single-cycle replicating viruses and their infection in HeLa-CD4-CCR5-β-Gal cells**. A). To evaluate the trans-incorporation of RT and IN in VSV-G pseudotyped viral particles, viruses released from 293T cells transfected with NLlucΔBglΔRI provirus alone (lane 6) or cotransfected with different Vpr-RT-IN expressors and a VSV-G expressor (lane 1 to 5) were lysed, immunoprecipitated with anti-HIV serum. Then, immunoprecipitates were run in 12% SDS-PAGE and analyzed by Western blot with rabbit anti-IN antibody (middle panel) or anti-RT and anti-p24 monoclonal antibody (upper and lower panel). B) The infectivity of trans-complemented viruses produced in 293 T cells was evaluated by MAGI assay. HeLa-CD4-CCR5-LTR-β-Gal cells were infected with equal amounts (at 10 cpm/cell) of different IN mutant viruses and after 48 hours of infection, numbers of β-Gal positive cells (infected cell) were monitored by X-gal staining. Error bars represent variation between duplicate samples and the data is representative of results obtained in three independent experiments.

Given that two di-lysine mutants located in the C-terminal domain of IN are involved in HIV-1 IN nuclear localization, we next evaluated whether these IN mutants would affect the efficiency of HIV-1 infection. To specifically analyze the effect of IN mutants in early steps of viral infection, we modified a previously described HIV-1 single-cycle replication system [32] and constructed a RT/IN/Env gene-deleted HIV-1 provirus NLlucΔBglΔRI, in which the *nef *gene was replaced by a firefly luciferase gene [33]. Co-expression of NLlucΔBglΔRI provirus with Vpr-RT-IN expressor and a vesicular stomatitis virus G (VSV-G) glycoprotein expressor will produce viral particles that can undergo a single-round of replication, since RT, IN and Env defects of provirus will be complemented *in trans *by VSV-G glycoprotein and Vpr-mediated RT and IN trans-incorporation [32]. This single cycle replication system allows us to introduce different mutations into IN gene sequence without differentially affecting viral morphogenesis and the activity of the central DNA Flap. After different IN mutations KK215,9AA, KK240,4AE and RR263,4AA were introduced into Vpr-RT-IN expressor, we produced VSV-G pseudotyped HIV-1 IN mutant virus stocks in 293T cells. In order to specifically investigate the effect of IN mutants on early steps during HIV-1 infection prior to integration, an IN class I mutant D64E was also included as control. After each viral stock was produced (as indicated in Fig. 3A), similar amounts of each virus stock (quantified by virion-associated RT activity) were lysed and virus composition and trans-incorporation of RT and IN of each virus stock were analyzed by Western blot analysis with anti-IN and anti-HIV antibodies, as described in Materials and Methods. Results showed that all VSV-G pseudotyped IN mutant viruses had similar levels of Gagp24, IN and RT, as compared to the wild-type virus (Fig. 3A), indicating that trans-incorporation of RT and IN as well as HIV-1 Gag processing were not differentially affected by the introduced IN mutations.

To test the infectivity of different IN mutant viruses in HeLa-CD4-CCR5-LTR-β-Gal cells, we first compared the infectivity of VSV-G pseudotyped wild type virus and the D64E mutant virus. At 48 hours post-infection with equivalent amount of each virus stock (at 1 cpm RT activity/cell), the number of β-Gal positive cells was evaluated by MAGI assay, as described previously [34]. Results showed that the number of infected cells (β-Gal positive cells) for D64E mutant reached approximately 14% of the wild type level (data not shown). This result is consistent with a previous report showing that, in HeLa MAGI assay, the infectivity level of class I IN integration-defect mutant was approximately 20 to 22% of wild type level [15]. It indicates that, even though the IN mutant D64E virus is defective for integrating viral DNA into host genome, *tat *expression from nucleus-associated and unintegrated viral DNAs can activate HIV-1 LTR-driven β-Gal expression in HeLa-CD4-CCR5-LTR-β-Gal cells. Indeed, several studies have already shown that HIV infection leads to selective transcription of *tat *and *nef *genes before integration [2,35,36]. Therefore, this HeLa-CD4-CCR5-LTR-β-Gal cell infection system provides an ideal method for us to evaluate the effect of different IN mutants on early steps of viral infection prior to integration. We next infected HeLa-CD4-CCR5-LTR-β-Gal cells with different VSV-G pseudotyped IN mutant viruses at higher infection dose of 10 cpm RT activity/cell and numbers of β-Gal positive cells were evaluated by MAGI assay after 48 hours of infection. Interestingly, results showed that the IN mutant D64E virus infection induced the highest level of β-Gal positive cells, whereas infection with viruses containing IN mutants KK215,9AA, KK240,4AE or RK263,4AA yielded much lower levels of β-Gal positive cells, which only reached approximately 11%, 5% or 26% of the level of D64E virus infection (Fig. 3B). Based on these results, we reasoned that these IN C-terminal mutants blocked infection mostly by affecting earlier steps of HIV-1 life cycle, such as reverse transcription and/or viral DNA nuclear import steps, which are different from the action of D64E mutant on viral DNA integration.

### Effect of IN mutants on viral infection in dividing and non-dividing C8166 T cells

**Figure 4:**
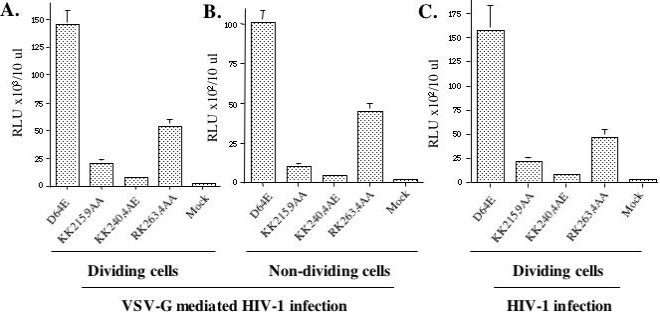
**Effect of IN mutants on viral infection in dividing and nondividing C8166 T cells**. To test the effect of different IN mutants on HIV-1 infection in CD4+ T cells, dividing (panel A) and non-dividing (aphidicolin-treated, panel B) C8166 T cells were infected with equal amount of VSV-G pseudotyped IN mutant viruses (at 5 cpm/cell). For evaluation of the effect of different IN mutants on HIV-1 envelope-mediated infection in CD4+ T cells, dividing C8166 T cells were infected with equal amount of HIV-1 envelope competent IN mutant viruses (at 10 cpm/cell) (panel C). After 48 hours of infection, HIV-1 DNA-mediated luciferase induction was monitored by luciferase assay. Briefly, the same amount (10^6 ^cells) of cells was lysed in 50 ul of luciferase lysis buffer and then, 10 μl of cell lysate was subjected to the luciferase assay. Error bars represent variation between duplicate samples and the data is representative of results obtained in three independent experiments.

To further test whether these C-terminal mutants could induce similar phenotypes in CD4^+ ^T cells, we infected dividing and non-dividing (aphidicolin-treated) C8166 CD4^+ ^T cells with equal amounts of VSV-G pseudotyped IN mutant viruses (at 5 cpm of RT activity/cell). Since all IN mutant viruses contain a luciferase (luc) gene in place of the *nef *gene, viral infection can be monitored by using a sensitive luc assay which could efficiently detect viral gene expression from integrated and unintegrated viral DNA [33]. After 48 hours of infection, equal amounts of cells were lysed in 50 μl of luc lysis buffer and then, 10 μl of cell lysates was used for measurement of luc activity, as described in Materials and Methods. Results showed that the D64E mutant infection in dividing C8166 T cells induced 14.3 × 10^4 ^RLU of luc activity (Fig. 4A), which was approximately 1000-fold lower than that in the wild type virus infection (data not shown). This level of luc activity detected in D64E mutant infection is mostly due to *nef *gene expression from the unintegrated DNA [33]. In agreement with the finding by MAGI assay described in figure 3, the Luc activity detected in KK215,9AA, KK240,4AE and RK263,4AA mutant samples were approximately 13%, 5% and 36% of level of D64E mutant infection (Fig. 4A). In parallel, infection of different IN mutants in non-dividing C8166 T cells was also evaluated and similar results were observed (Fig. 4B).

To test whether these IN mutants had similar effects during HIV-1 envelope-mediated single cycle infection, we produced virus stocks by co-transfecting 293T cells with a HIV-1 envelope-competent NLlucΔRI provirus with each Vpr-RT-IN mutant expressor, as described in Materials and Methods. Then, dividing CD4^+ ^C8166 cells were infected with each virus stock (at 10 cpm RT activity/cells). At 48 hours post-infection, cells were collected and measured for luc activity. Results from figure 4C showed that, similar to results obtained from VSV-G pseudotyped virus infection (Fig. 4A), the Luc activity detected in cells infected by HIV-1 envelope competent KK215,9AA, KK240,4AE and RK263,4AA mutant viruses were approximately 13.5%, 6% and 29% of level of D64E mutant infection (Fig. 4C). All of these results confirm the data from HeLa-CD4-CCR5-LTR-β-Gal infection (Fig. 3) by using either VSV-G- and HIV-1 envelope-mediated infections and suggest again that the significantly attenuated infection of KK215,9AA, KK240,4AE and RK263,4AA mutant viruses may be due to their defect(s) at reverse transcription and/or viral DNA nuclear import steps.

### Effects of IN mutants on reverse transcription, viral DNA nuclear import and integration

**Figure 5:**
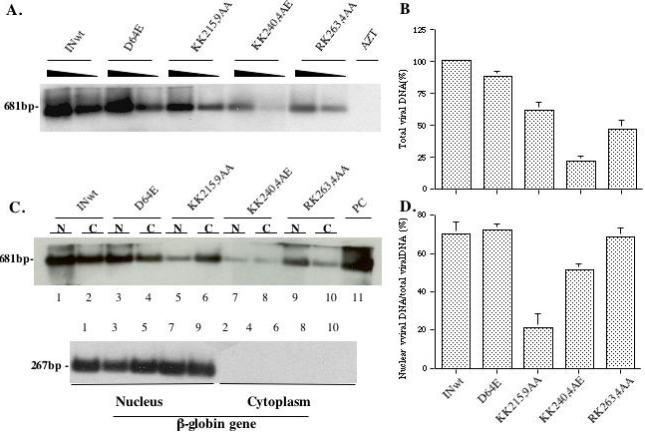
**Effects of different IN mutants on HIV-1 reverse transcription and DNA nuclear import**. Dividing C8166 T cells were infected with equal amounts of different HIV-1 IN mutant viruses. A) At 12 hours post-infection, 1 × 10^6 ^cells were lysed and the total viral DNA was detected by PCR using HIV-1 LTR-Gag primers and Southern blot. B) Levels of HIV-1 late reverse transcription products detected in panel A were quantified by laser densitometry and viral DNA level of the wt virus was arbitrarily set as 100%. Means and standard deviations from two independent experiments are presented. C) At 24 hours post-infection, 2 × 10^6 ^cells were fractionated into cytoplasmic and nuclear fractions as described in Materials and Methods. The amount of viral DNA in cytoplasmic and nuclear fractions were analyzed by PCR using HIV-1 LTR-Gag primers and Southern blot (upper panel, N. nuclear fraction; C. cytoplasmic fraction). Purity and DNA content of each subcellular fraction were monitored by PCR detection of human globin DNA and visualized by specific Southern blot (lower panel). D). The percentage of nucleus-associated viral DNA relative to the total amount of viral DNA for each mutant was also quantified by laser densitometry. Means and standard deviations from two independent experiments are shown.

All results so far suggest that these C-terminal mutants might significantly affect early steps during HIV-1 replication. To directly assess the effect of these IN C-terminal mutants on each early step during viral infection, we analyzed the viral DNA synthesis, their nuclear translocation and integration following each IN mutant infection in dividing C8166 cells. Levels of HIV-1 late reverse transcription products were analyzed by semi-quantitative PCR after 12 hours of infection with HIV-1 specific 5'-LTR-U3/3'-Gag primers and Southern blot, as previously described [32,37]. Also, intensity of amplified HIV-1 specific DNA in each sample was evaluated by laser densitometric scanning of bands in Southern blot autoradiograms (Fig. 5A). Results showed that total viral DNA synthesis in both KK215,9AA and RK263,4AA infection reached approximately 61% and 46% of that of the wild type (wt) virus infection (Fig. 5A and 5B). Strikingly, in KK240,4AA sample, detection of viral DNA synthesis was drastically reduced, which only reached 21% of viral DNA level in WT sample (Fig. 5A and 5B). These results indicate that all three C-terminal mutants negatively affected viral reverse transcription during viral infection and KK240,4AA mutant exhibited most profound effect.

Meanwhile, the nucleus- and cytoplasm-associated viral DNA levels were analyzed at 24 hours post-infection in C8166 T cells. The infected cells were first gently lysed and separated into nuclear and cytoplasmic fractions by using a previously described fractionation technique [37]. Then, levels of HIV-1 late reverse transcription products in each fraction were analyzed by semi-quantitative PCR, as described above. Results revealed differential effects of C-terminal mutants on HIV-1 DNA nuclear import. In the wt, D64E and RK263,4AA virus-infected samples, there were respectively 70%, 72% and 68% of viral DNA associated with nuclear fractions (Fig. 5C (upper panel, lanes 1 and 2; 3 and 4; 9 and 10) and 5D). For KK240,4AE mutant, approximately 51% of viral DNA was nucleus-associated (Fig. 5C (upper panel, lane 7 and 8) and 5D). Remarkably, in KK215,9AA infected sample, viral cDNA was found predominantly in the cytoplasm and only approximately 21% of viral DNA was associated with the nuclear fraction (Fig. 5C (upper panel, lane 5 and 6) and 5D). Meanwhile, the integrity of fractionation procedure was validated by detection of β-globin DNA, which was found solely in the nucleus and levels of this nucleus-associated cellular DNA were similar in each nuclear sample (Fig. 5C, lower panel).

**Figure 6:**
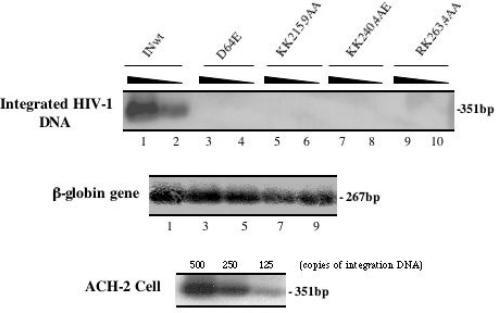
**Effect of IN mutants on HIV-1 proviral DNA integration**. Dividing C8166 T cells were infected with equal amounts of different HIV-1 IN mutant viruses. At 24 hours post-infection, 1 × 10^6 ^cells were lysed and serial-diluted cell lysates were analyzed by two-step Alu-PCR and Southern blot for specific detection of integrated proviral DNA from infected cells (Upper panel). The DNA content of each lysis sample was also monitored by PCR detection of human β-globin DNA and visualized by specific Southern blot (middle panel). The serial-diluted ACH-2 cell lysates were analyzed for integrated viral DNA and as quantitative control (lower panel). The results are representative for two independent experiments.

Even though the C-terminal mutants were shown to significantly affect HIV-1 reverse transcription and/or nuclear import, the various low levels of nucleus-associated viral DNA during the early stage of replication (Fig. 5C) may still be accessible for viral DNA integration. To address this question, 1 × 10^6 ^dividing C8166 T cells were infected with equivalent amounts of each single cycle replicating virus stock (5 cpm/cell), as indicated in figure 6 and after 24 hours of infection, the virus integration level was checked by using a previously described sensitive Alu-PCR technique [32], Results revealed that, while the wt virus resulted in an efficient viral DNA integration (Fig. 6, upper panel; lanes 1 and 2), there was no viral DNA integration detected in D64E mutant (lanes 3 to 4) and in all three C-terminal mutant infection samples (lanes 5 to 10), although similar levels of cellular β-globin gene were detected in each sample (Fig. 6, middle panel). These results suggest that, in addition to affecting HIV-1 reverse transcription and nuclear import, all three C-terminal IN mutants tested in this study also negatively affected viral DNA integration. Overall, all of these results indicate that all three IN C-terminal mutants are belonged to class II mutants, which affected different early steps during HIV-1 replication. Among these mutants, the KK240,4AE showed the most profound inhibition on reverse transcription and the KK215,9AA, and to a lesser extent, KK240,4AE, impaired viral DNA nuclear translocation during early HIV-1 infection in C8166 T cells.

## Discussion

In this study, we performed mutagenic studies to analyze different regions in the C-terminal domain of HIV-1 IN that contribute to protein nuclear localization as well as their effects on virus infection. First, our analyses showed that specific lysine mutations introduced in two highly conserved tri-lysine regions in the C-terminal domain of HIV-1 IN impaired protein nuclear accumulation. Second, infection experiments revealed that all three C-terminal mutant viruses (KK215,9AA, KK240,4AE and RK263,4AA) exhibited more severe defect of induction of β-Gal positive cells and luc activity, as compared to an IN class 1 mutant D64E virus, in CD4^+ ^HeLa-β-Gal cells, dividing and non-dividing C8166 T cells. It suggests that all three C-terminal mutant virus infections may have defects at steps prior to integration. Further analysis of total viral DNA synthesis, viral DNA nuclear import and integration indicates that all three C-terminal mutants displayed a class II mutant profile. Even though all of them reduced viral reverse transcription levels, the mutant KK240,4AE showed the most profound inhibitory effect. In addition, the mutant KK215,9AA, and to a lesser extent, KK240,4AE, impaired viral DNA nuclear translocation. These IN mutant-induced defects do not appear to result from various effects of mutants on Gag-Pol processing and maturation given that RT and IN were complemented *in trans *in this HIV-1 single-cycle infection system. Rather, the effect of different IN mutants on reverse transcription and viral DNA nuclear import is likely originated from a role of mutants within the maturing PIC complexes.

Previous work by Gallay *et al*., have proposed an atypical bipartite NLS (^186^KRK and ^211^KELQKQITK) in HIV-1 IN by finding that IN mutants K186Q and Q214/216L lost their karyophilic feature and their ability to bind to karyopherin α *in vitro *[3]. Even though these results were confirmed by Petit and colleagues by studying the intracellular localization of HIV-1 Flag-IN [18], other studies, using GFP-IN fusion protein, did not reveal the importance of K186Q and Q214/216L mutations for HIV-1 IN nuclear localization [16,23,27]. Therefore, the definition of region(s) in HIV-1 IN contributing to the protein nuclear localization is still controversial. In this study, we investigated the intracellular localization of several IN-YFP fusion proteins including the C-terminal-deletion mutant IN_1–212_-YFP, substitution mutants IN_KK215,9AA_-YFP and IN_KK240,4AE_-YFP and found that all of these IN fusion mutants impaired protein nuclear accumulation. It suggests that two C-terminal tri-lysine regions ^211^KELQKQITK and ^236^KGPAKLLWK contribute to IN nuclear localization. Interestingly, the study by Maertens *et al *also showed that the fusion of HIV-1 IN C-terminal fragment alone with GFP rendered fusion protein to be exclusively in the nucleus, speculating that the C-terminal domain may have a role in HIV-1 nuclear import [28]. However, at this moment, we still could not exclude the possibility that the IN nuclear accumulation could be facilitated by the DNA binding ability of IN protein, as suggested by Devroe *et al *[27]. It has to be noted that two studies have previously observed the nuclear localization of GFP-IN fusion proteins although the C-terminal domain of IN was deleted from the fusion protein [23,28]. It has also been shown that both N-terminal zinc binding domain and the central core domain of HIV-1 IN are involved in its interaction with a cellular protein, human lens epithelium-derived growth factor/transcription coactivator p75 (LEDGF/p75) and this IN/LEDGF/p75 interaction is required for GFP-IN nuclear localization [28]. However, our deletion analysis by using IN-YFP fusion protein failed to reveal the importance of both N-terminal and core domains for IN nuclear localization (Fig. 1). One explanation for this discrepancy could be different orientations of fusion proteins used in our study (IN-YFP) and other studies (GFP-IN). It is possible that different forms of fusion proteins may differentially affect the ability of IN to interact with LEDGF/p75 and consequently affect their ability for nuclear targeting. Therefore, it would be interesting to test whether IN_KK215,9AA_-YFP and IN_KK240,4AE_-YFP could loss their ability to interact with LEDGF/p75. These studies are underway.

An important question that needs to be addressed is the impact of nuclear localization-defective IN mutants on HIV-1 replication. Given that most IN mutants characterized so far are classified as class II mutants that cause pleiotropic damage including defects in viral morphogenesis, reverse transcription and integration [16,38], we used a previously described VSV-G pseudotyped HIV-1 RT/IN trans-complement single-cycle replication system [32,39] to minimize differential effects of IN mutants on virus maturation. Also, in our infection experiments, a specific integration-defective class I mutant D64E virus was introduced in order to monitor the viral gene expression from unintegrated HIV-1 DNA species that are already translocated into nucleus during virus infection. It is known that certain levels of selected viral gene expression (*tat and nef*) from unintegrated viral DNA species are detected during this Class I mutant infection [2,35,36]. Interestingly, our infection analysis revealed that more profound infection defects were found for all three IN C-terminal mutant viruses KK215,9AA, KK240,4AE and RK263,4AA than D64E mutant virus in Hela-CD4-CCR5-β-Gal cells, dividing and non-dividing C8166 T cells (Fig. 3 and 4). These results suggest that these C-terminal IN mutants may affect early steps such as reverse transcription and/or nuclear import and consequently result in a reduced level of viral DNA in the nucleus, which is accessible for *tat *and *nef *expression, To understand the mechanism(s) underlying replication defects of each C-terminal mutant, levels of total reverse transcription were analyzed during early viral infection. Consistent with a previous study [6], infection with D64E mutant virus did not affect reverse transcription as compared to wt virus infection. However, all three C-terminal mutants display various levels of impaired HIV-1 reverse transcription (Fig. 5A and 5B). The mutant KK240,4AE showed strongest inhibition of reverse transcription (21% compared to the wt level (100%)), while mutants KK215,9AA and RK263,4AA reached to 61% and 46% (Fig. 5A and 5B). These data indicate that all of these IN mutants, especially KK240,4AA, negatively affect reverse transcription at early viral infection. Consistently, recent studies have shown that the C-terminal domain of IN contributes to efficient reverse transcription and this domain of IN was able to bind to heterodimeric RT [6,40,41]. It is possible that these C-terminal mutants, especially for KK240,4AE, may disrupt the interaction between IN and RT and result in decreased viral cDNA synthesis.

Subsequently, we examined levels of nucleus- and cytoplasm-associated viral DNA during early virus infection. Results clearly show that the nuclear localization defective mutant KK215,9AA leads to significantly reduced levels of viral DNA in the nucleus, as compared to the wt and D64E viruses (Fig. 5C and 5D). It suggests that the Q region is in fact important for HIV-1 nuclear import. Consistently, a recent study by Lu *et al *also observed that infection of K215A/K219A mutant induced more than 3-fold lower luc activity compared to class I IN mutant D64N/D116N [16]. Moreover, similar to our experimental system, their study revealed that, in the context of VSV-G pseudotyped virus infection in Jurkat cells, 2-LTR circle DNA levels of K215A/K219A and Q214L/Q216L were significantly lower than other mutants V165A and C130G, even though the inhibition of viral reverse transcription mediated by these mutants were comparable [16]. In addition, KK240,4AE mutant also showed a modest impairment of viral DNA nuclear import (Fig. 5C and 5D). In fact, this mutant exhibited the most profound infection defect, compared to other two mutants (KK215,9AA and RK263,4AA) (Fig. 3 and 4). This may be due to combined effects of this mutant on both reverse transcription and viral DNA nuclear import, as shown in Fig. 5. One interesting question is whether such profound infection defect of KK240,4AE mutant virus could be due to a structural alteration by replacing glutamic acid (E) for lysine at position of 244. It seems to be unlikely since 1) the effect of this mutant on nuclear import was not as dramatic as KK215,9AA mutant (as shown in Fig. 5); 2) Wiskerchen *et al *have reported that infection of MAGI cells with two other IN mutants K236A/K240A and K244A/E246A mutants, that are located in the same region as our KK240,4AE mutant, resulted in 0 and 4 β-Gal positive cells, while infection of class I IN mutants produced 700 to 1400 β-Gal positive cells [15]. All of these observations suggest that this region indeed plays an important role for IN activities during early stage of virus infection prior to integration. Also, it has to be noted that although similar inhibition of reverse transcription was seen for KK215,9AA and RK263,4AA mutants, RK263,4AA mutant induced two to three fold higher level of β-Gal positive cells and luc activity than KK215,9AA mutant (Fig. 3 and 4). This is expected since KK215,9AA affected both reverse transcription and nuclear import, while RK263,4AA mutant only impaired reverse transcription (Fig. 5). In addition, our analysis could not detect viral DNA integration in each C-terminal mutant infection (Fig. 6), even though they displayed various low levels of nucleus-associated viral DNA (Fig. 5C). It suggests that these IN mutants may also negatively affect viral integration during their infection. Alternatively, it could be possible that these mutants may have additional defect(s) at an undefined postnuclear entry step that is required for viral DNA integration, as suggested by Lu *et al *[16]. Consistently, their recent reports have shown that several IN mutants in same regions, including K215A/K219A, E244A and R262A/K264A, completely lost virus replication ability in CD4+ Jurkat T cells [16,42].

Up to now, the mechanism(s) underlying the action of HIV-1 IN in viral PIC nuclear import is still unclear. Since IN is a component of viral PIC, at least two factors may affect the contribution of IN to viral PIC nuclear import: first, IN needs to directly or indirectly associate with viral DNA and/or other PIC-associated proteins in order to participate in driving viral DNA into the nucleus; second, IN needs to have a NLS and/or bind to other karyophilic proteins for nuclear translocation. Any mutation disrupting one of these two abilities would affect IN's action for viral DNA nuclear import. A recent study evaluated the effect of several IN core domain mutants targeting key residues for DNA recognition on HIV-1 replication and indicated that, while all of these IN mutants maintained their karyophilic properties, viruses harboring these mutants still severely impaired viral DNA nuclear import [4]. In our study, both KK215,9AA and KK240,4AE mutants clearly lost their karyophilic properties and negatively affected viral DNA nuclear import. However, it is still premature to define these regions acting as IN NLS, even though a previously described IN mutant Q214/216L, which is also located in proximal tri-lysine domain, has been shown to reduce IN-karyopherin α interaction *in vitro *[3]. More studies are required for further characterization of molecular mechanisms underlying the action of these IN mutants during HIV-1 DNA nuclear import.

## Conclusion

Taken together, the results presented here highlight that all three C-terminal mutants tested in this study resulted in drastic loss of viral infectivity that were due to defects in different early steps of viral replication. Specific lysine mutations introduced in the tri-lysine regions of the C-terminal domain of HIV-1 IN, especially for KK215,9AA, impaired protein nuclear accumulation and HIV-1 PIC nuclear import. Although all of C-terminal mutants inhibited viral reverse transcription to different extents, KK240,4AE mutant exhibited most profound effect on this step. These results suggest that the tri-lysine regions (^211^KELQKQITK and ^236^KGPAKLLWK) in the C-terminal of IN are important for HIV-1 reverse transcription and/or nuclear import. More studies are underway to further characterize the mechanisms involved in the action of these regions during early steps of HIV-1 replication.

## Materials and methods

### Construction of different IN expressors and HIV-1 RT/IN defective provirus

The full-length wild-type HIV-1 IN cDNA was amplified by polymerase chain reaction (PCR) using HIV-1 HxBru strain [30] as template and an engineered initiation codon (ATG) was placed prior to the first amino acid (aa) of IN. The primers are 5'-IN-HindIII-ATG (5'-GCGCAAGCTTGGATAGATGTTTTTAGATGGAA-3') and 3'-IN-Asp718 (5'-CCATGTGTGGTACCTCATCCTGCT-3'). The PCR product was digested with *Hind*III and *Asp*718 restriction enzymes and cloned in frame to 5' end of EYFP cDNA in a pEYFP-N1 vector (BD Biosciences Clontech) and generated a IN-YFP fusion expressor. Also, cDNA encoding for truncated IN (aa 50 to 288 or aa 1 to 212) was amplified by PCR and also cloned into pEYFP-N1 vector. The primers for generation of IN50-288 cDNA are IN50-HindIII-ATG-5'(5'– GCGCAAGCTTGGATAGATGCATGGACAAGTAG-3) and 3'-IN-Asp718 and primers for amplifying IN1-212 cDNA are IN-HindIII-ATG-5' and IN-212-XmaI-3'(5'-CAATTCCCGGGTTTGTATGTCTGTTTGC-3). IN substitution mutants IN_KK215,9AA_-YFP, IN_KK240,4AE_-YFP and IN_RK263,4AA_-YFP, were generated by a two-step PCR-based method [43] by using a 5'-primer (5'-IN-HindIII-ATG), a 3'-primer (3'-IN-Asp718) and complementary primers containing desired mutations. Amplified IN cDNAs harboring specific mutations were then cloned into pEYFP-N1 vector. To improve the expression of each IN-YFP fusion protein, all IN-YFP fusing cDNAs were finally subcloned into a SVCMV vector, which contains a cytomegalovirus (CMV) immediate early gene promoter [43].

To construct HIV-1 RT/IN defective provirus NLlucΔBglΔRI, we used a previously described HIV-1 envelope-deleted NLlucΔBglD64E provirus as the backbone (kindly provided by Dr. Irvin S.Y. Chen). In this provirus, the *nef *gene was replaced by a firefly luciferase gene [33]. The *Apa*I/*Sal*I cDNA fragment in NLlucBglD64E was replaced by the corresponding fragment derived from a HIV-1 RT/IN deleted provirus R^-^/ΔRI [32] and generated a RT/IN deleted provirus NLlucΔBglΔRI, in which RT and IN gene sequences were deleted while a 194-bp sequence harboring cPPT/CTS *cis*-acting elements was maintained. To restore HIV-1 envelope gene sequence in NLlucΔBglΔRI provirus, the *Sal*I/*BamH*I cDNA fragment in this provirus was replaced by a corresponding cDNA fragment from a HIV-1 envelope competent provirus R^-^/ΔRI [32] and the resulting provirus is named as NLlucΔRI. To functionally complement RT/IN defects of NLlucΔBglΔRI, a CMV-Vpr-RT-IN fusion protein expressor [32] was used in this study. Co-transfection of NLlucΔBglΔRI, CMV-Vpr-RT-IN and a vesicular stomatitis virus G (VSV-G) glycoprotein expressor results in the production of VSV-G pseudotyped HIV-1 that can undergo for single cycle replication in different cell types [32]. To investigate the effect of IN mutants on viral replication, different mutants KK215,9AA, KK240.4AE, RK263,4AA or D64E were introduced into CMV-Vpr-RT-IN expressor by PCR-based method as described above and using a 5'-primer corresponding to a sequence in RT gene and including a natural *Nhe*I site (5'-GCAGCTAGCAGGGAGACTAA-3'), a 3'-primer (3'-IN-stop-*Pst*I, 5'– CTGTTCCTGCAGCTAATCCTCATCCTG-3') and the complementary oligonucleotide primers containing desired mutations. All IN mutants were subsequently analyzed by DNA sequencing to confirm the presence of mutations or deletions.

### Cell lines and reagents

Human embryonic kidney 293T, HeLa and HeLa-CD4-CCR5-β-Gal cells were maintained in Dulbecco's Modified Eagles Medium (DMEM) supplemented with 10% fetal calf serum (FCS). Human C8166 T-lymphoid cells were maintained in RPMI-1640 medium. Antibodies used in the immunofluorescent assay, immunoprecipitation or western blot are as follows: The HIV-1 positive human serum 162 and anti-HIVp24 monoclonal antibody used in this study were previously described [44]. The rabbit anti-GFP and anti-IN antibodies were respectively obtained from Molecular Probes Inc and through AIDS Research Reference Reagent Program, Division of AIDS, NIAID, NIH. Aphidicolin was obtained from Sigma Inc.

### Cell transfection and immunofluorescence assay

DNA transfection in 293T and HeLa cells were performed with standard calcium phosphate DNA precipitation method. For immunofluorescence analysis, HeLa cells were grown on glass coverslip (12 mm^2^) in 24-well plate. After 48 h of transfection, cells on the coverslip were fixed with PBS-4% paraformaldehyde for 5 minutes, permeabilized in PBS-0.2% Triton X-100 for 5 minutes and incubated with primary antibodies specific for GFP or HIV-1 IN followed by corresponding secondary FITC-conjugated antibodies. Then, cells on the coverslip were viewed using a computerized Axiovert 200 inverted fluorescence microscopy (Becton Deckson Inc).

### Virus production and infection

Production of different single-cycle replicating virus stocks and measurement of virus titer were previously described [32]. Briefly, 293T cells were co-transfected with RT/IN defective NLlucΔBglΔRI provius, a VSV-G expressor and each of CMV-Vpr-RT-IN (wt/mutant) expressor. To produce HIV-1 envelope competent single cycle replicating virus, 293T cells were co-transfected with NLlucΔRI and different CMV-Vpr-RT-IN (wt/mutant) expressors. After 48 hours of transfection, supernatants were collected and virus titers were quantified by RT activity assay [43].

To test the effect of IN mutants on virus infection, equal amounts of virus were used to infect HeLa-CCR5-CD4-β-Gal cells, dividing and non-dividing C8166 T cells. To compare the infection of each viral stock in HeLa-CCR5-CD4-β-Gal cells, numbers of infected cells (β-Gal positive cells) were evaluated by the MAGI assay 48 hours post-infection (p.i) as described previously [34]. To infect CD4+ T cells, dividing or aphidicolin-treated non-dividing C8166 T cells (with 1.3 μg/ml of aphidicolin) were infected with equivalent amounts of single cycle replicating viruses (5 cpm/cell) for 2 hours. Then, infected cells were washed and cultured in the absence or presence of the same concentration of aphidicolin. At 48 hours post-infection, 1 × 10^6 ^cells from each sample were collected, washed twice with PBS, lysed with 50 μl of luciferase lysis buffer (Fisher Scientific Inc) and then, 10 μl of cell lysate was subjected to the luciferase assay by using a TopCount^®^NXT™ Microplate Scintillation & Luminescence Counter (Packard, Meriden) and the luciferase activity was valued as relative luciferase units (RLU). Each sample was analyzed in duplicate and the average deviation was calculated.

### Immunoprecipitation and Western blot analyses

For detection of IN-YFP fusion proteins, 293T cells transfected with each IN-YFP expressor were lysed with RIPA lysis buffer and immunoprecipitated using human anti-HIV serum. Then, immunoprecipitates were run in 12% SDS-PAGE and analyzed by Western blot using rabbit anti-GFP antibody. To analyze virion-incorporation of IN and virus composition, 293T cells were co-transfected with NLlucΔBglΔRI provirus and each of CMV-Vpr-RT-IN (wt/mutant) expressors. After 48 hours, viruses were collected, lysed with RIPA lysis buffer and immunoprecipitated with human anti-HIV serum. Then, immunoprecipitates were run in 12% SDS-PAGE and analyzed by Western blot with rabbit anti-IN antibody and anti-p24 monoclonal antibody or anti-HIV serum.

### HIV-1 reverse-transcribed and integrated DNA detection by PCR and Southern blotting

C8166 T cells were infected with equal amount of the wt or IN mutant viruses for 2 hours, washed for three times and cultured in RPMI medium. To detect total viral DNA synthesis, at 12 hours post-infection, equal number (1 × 10^6 ^cells) of cells were collected, washed twice with PCR washing buffer (20 mM Tris-HCl, pH8.0, 100 mM KCl), and lysed in lysis buffer (PCR washing buffer containing 0.05% NP-40, 0.05% Tween-20). Lysates were then incubated at 56°C for 30 min with proteinase K (100 μg/ml) and at 90°C for 10 min prior to phenol-chloroform DNA purification. To detect viral cDNA from each sample, all lysates were serially diluted 5-fold and subjected to PCR analysis. The primers used to detect late reverse transcription products were as following: 5'-LTR-U3, 5'-GGATGGTGCTTCAAGCTAGTACC-3' (nt position 8807, +1 = start of BRU of transcription initiation); 3'-Gag 5'-ACTGACGCTCTCGCACCCATCTCTCTC-3' (nt position 329). The probe for southern blot detection was generated by PCR with a 5'-LTR-U5 oligonucleotide, 5'-CTCTAGCAGTGGCGCCCGAACAGGGAC-3' (nt position 173) and the 3'-Gag oligo. PCR was carried out using 1× HotStar Taq Master Mix kit (QIAGEN, Mississauga, Ontario), as described previously [32].

To analyze nucleus- and cytoplasm-associated viral DNA, a subcellular fractionation of infected C8166 T cells (2 × 10^6^) was performed after 24 hours of infection, as described previously [37]. Briefly, infected cells were pelleted and resuspended in ice-cold PCR lysis buffer (washing buffer containing 0,1% NP-40). After a 5-min incubation on ice, the nucleus was pelleted by centrifugation, washed twice with PCR wash buffer, and lysed in lysis buffer (0,05% NP-40, 0,05% Tween-20). Then, both cytoplasmic sample (supernatant from the first centrifugation) and the nuclear sample were treated with proteinase K and used for PCR analysis, as described above.

Integrated proviral DNA was detected in cell lysates by a modified nested Alu-PCR [32], in which following the first PCR, a second PCR was carried-out to amplify a portion of the HIV-1 LTR sequence from the first Alu-LTR PCR-amplified products. The first PCR was carried out by using primers including 5'-Alu oligo (5'-TCCCAGCTACTCGGGAGGCTGAGG-3') and 3'-LTR oligo (5'-AGGCAAGCTTTATTGAGGGCTTAAGC-3') (nt position 9194) located respectively in the conserved region of human Alu sequence and in HIV-1 LTR. The primer used for both of the second nested PCR and for generating a probe are 5'-NI: 5'-CACACACAAGGCTACTTCCCT-3' and 3'-NI: 5'-GCCACTCCCCAGTCCCGCCC-3'. As a control, the first and second PCR primer pairs were also used in parallel to detect integrated viral DNA from serially diluted ACH-2 cells, which contain one viral copy/cell, in a background of uninfected C8166 cellular DNA.

To evaluate the DNA content of extracted chromosomal DNA preparations, detection of human β-globin gene was carried-out by PCR, as described previously [37]. All final PCR products were electrophoresed through 1.2% agarose gel and transferred to hybridization transfer membrane (GeneScreen Plus, PerkinElmer Life Sciences), subjected to Southern hybridization by using specific PCR DIG-Labeling probes (Roche Diagnostics, Laval, Que) and visualized by a chemiluminescent method. Densitometric analysis was performed using a Personal Molecular Imager (Bio-Rad) and Quantity One software version 4.1.

## Authors' contributions

Z-J Ao designed and performed experiments, constructed most IN mutants and wrote the manuscript. KR Fowke provided technique support and critically evaluated the manuscript. EA Cohen participated in the design of the study and critically evaluated the manuscript. X-J Yao designed the study and coordinated it. All authors read and approved the final manuscript.
